# Revealing hidden medium-range order in amorphous materials using topological data analysis

**DOI:** 10.1126/sciadv.abc2320

**Published:** 2020-09-09

**Authors:** Søren S. Sørensen, Christophe A. N. Biscio, Mathieu Bauchy, Lisbeth Fajstrup, Morten M. Smedskjaer

**Affiliations:** 1 Department of Chemistry and Bioscience, Aalborg University, DK-9220 Aalborg, Denmark.; 2 Department of Mathematical Sciences, Aalborg University, DK-9220 Aalborg, Denmark.; 3 Physics of AmoRphous and Inorganic Solids Laboratory (PARISlab), Department of Civil and Environmental Engineering, University of California, Los Angeles, Los Angeles, CA 90095, USA.

## Abstract

Despite the numerous technological applications of amorphous materials, such as glasses, the understanding of their medium-range order (MRO) structure—and particularly the origin of the first sharp diffraction peak (FSDP) in the structure factor—remains elusive. Here, we use persistent homology, an emergent type of topological data analysis, to understand MRO structure in sodium silicate glasses. To enable this analysis, we introduce a self-consistent categorization of rings with rigorous geometrical definitions of the structural entities. Furthermore, we enable quantitative comparison of the persistence diagrams by computing the cumulative sum of all points weighted by their lifetime. On the basis of these analysis methods, we show that the approach can be used to deconvolute the contributions of various MRO features to the FSDP. More generally, the developed methodology can be applied to analyze and categorize molecular dynamics data and understand MRO structure in any class of amorphous solids.

## Teaser

Hidden medium-range structural features of disordered materials can be extracted through persistent homology analyses.

## INTRODUCTION

Characterization and understanding of medium-range order (MRO) structure of amorphous materials continues to be an active area of research (*1*–*4*). In addition to offering new fundamental insights into the glassy state, such research is needed for explaining the variation in various glass properties (*5*). Typically, MRO in covalent glasses is regarded as structural features in the range of 5 to 20 Å, including arrangements of connected polyhedra, superstructural units, and clustering around structural features such as network modifiers (*6*). Neutron and x-ray diffraction techniques have been widely used to characterize MRO structure of glasses by determining the structure factor, *S*(*Q*). However, the structural interpretation of *S*(*Q*) is nontrivial because of its origin in reciprocal space, and as a result, the origin of the first sharp diffraction peak (FSDP) in *S*(*Q*) has been intensively debated. The FSDP has been associated with various anomalies (*6*, *7*), including anomalous temperature (*8*, *9*), pressure (*10*, *11*), and composition behavior (*12*, *13*).

While the relation of the FSDP with MRO structure is widely accepted, numerous structural origins have been assigned to the FSDP. In glassy silicates, the FSDP has been proposed to originate from quasicrystalline and ring-type structures similar to those found in crystalline silica (*3*, *14*). Similarly, for chalcogenide glasses, the FSDP has been assigned to layer-like structures (*8*, *9*) or cluster-like regions with well-defined periodicities (*15*, *16*). The latter interpretation is also related to Elliot’s void model, ascribing the anomalous temperature, pressure, and composition behavior of the FSDP to voids around cation-centered clusters explained by a prepeak in the concentration-concentration structure factor [*S*_CC_(*Q*)] of the Bhatia-Thornton formalism (*6*). This model has been successfully applied to various amorphous systems and has also explained some of the anomalous behavior of the FSDP (*6*). Besides experimental diffraction measurements, molecular dynamics simulations have deepened the understanding of the FSDP and MRO structure, owing to their ability to provide real-space structural information. For example, using first-principles molecular dynamics simulations, the interpretation of Elliot has been questioned based on the lacking FSDP in a model GeSe_2_ liquid, despite the presence of a prepeak in its *S*_CC_(*Q*) (*6*, *17*). Similarly, no experimental prepeak in the *S*_CC_(*Q*) in vitreous SiO_2_ has been identified based on neutron diffraction experiments (*18*). For alkali silicates, the compositional dependence of the FSDP can be reproduced by classical molecular dynamics simulations, ascribing most of the FSDP anomalies to scattering length differences (*12*, *13*, *19*). More recently, the FSDP in various tetrahedrally coordinated glasses has been ascribed to be a signature of the local ordering of the tetrahedral unit itself (*4*).

**Fig. 1:**
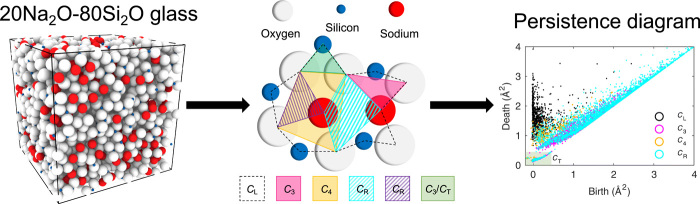
Schematic overview of the process of obtaining a persistence diagram. Here, the trajectory information for a 20Na_2_O-80SiO_2_ glass at 300 K (oxygen, white; silicon, blue; and sodium, red) is used to construct the persistence diagram. The persistence diagram coloring represents the characteristic regions, with the note that the turquoise points represent both the turquoise and violet *C*_R_ regions. Stripes indicate that the region is fully or partially overlapping with another region.

Recently, persistent homology, a type of topological data analysis, has been proposed as a tool to describe the MRO structure of SiO_2_ glass (*20*, *21*), CuZr and Pd_40_Ni_40_P_20_ metallic glasses (*21*, *22*), and amorphous ices (*23*). Persistent homology is a novel approach to analyze high-dimensional datasets, by focusing on the global properties such as data shape and connectivity, with possible applications, e.g., in biology, physics, and chemistry (*24*). In the case of glass structure, atomic trajectories generated from molecular dynamics simulations and the atomic radii are used as input to generate persistence diagrams, which are two-dimensional (2D) histograms of both the fraction and scale of the structural features, most importantly, rings and cavities embedded in the atomic configuration (Fig. 1). As an advantage compared to standard ring size analyses, persistent homology is not limited to chemically bonded structures but provides a fingerprint of the entire arrangement of atoms.

In topological data analysis, and here specifically persistent homology, tools from the mathematical area of algebraic topology are used to extract features such as components, rings, and voids from a shape built from a given network of data points. In the present case, the nodes of the network are the positions of the atoms, which are distributed in the Euclidean space. The procedure for obtaining the persistence diagram of such atomic network is as follows. First, a ball replaces each atom based on its radius. The radius of each ball is then gradually increased with the same increment for all atom types. Following the study by Hiraoka *et al*. (*21*), the increment is an increment in the radius squared, and as in the work of Carlsson (*25*) and Biscio and Møller (*26*), we refer to this increment as “time.” For calculations, the union of balls is actually described as a union of more complicated convex shapes, which intersect less than the original balls, making computations feasible by giving fewer edges, triangles, and tetrahedra. This construction is equivalent to the union of balls only when the increment of the radius is squared, as described in detail elsewhere (*27*).

This procedure of continuously increasing radii is used to build a sequence of structures with (i) a point for each atom, (ii) an edge between two points when the corresponding growing balls intersect for the first time, (iii) a triangle is filled in when three balls have a common intersection, and (iv) similarly for tetrahedra, etc. The time at which the addition of an edge gives rise to a closed loop is denoted as “birth,” while the time at which the addition of a filled triangle gives the first structure bounded by the loop is the “death” of the loop. Similarly, a void is born when the addition of a triangle gives rise to a surface enclosing the void. It dies when a tetrahedron causes the void to be filled. On the other hand, in the simplest case of persistent homology, all connected components are born simultaneously (*b* = 0) and die when an edge between two points intersect for the first time. This gives rise to a persistence diagram, a scatterplot of death versus birth, essentially holding the same information as the radial distribution function (RDF). Such analysis is referred to as 0D persistent homology. Analyses of loops and voids are then denoted as 1D and 2D persistent homology, respectively. In this work, we focus on categorizing loops and voids and use a loop definition based on the first appearance of edges. In the description of the RDF, we rely on the definition of Keen (*28*), while the structure factors are calculated on the basis of the Faber-Ziman formalism (*29*).

In the case of amorphous materials, the loops take the form of geometrical rings of atoms, which are not necessarily covalently bonded. As such, the persistent homology approach differs from traditional ring analysis (*30*). While the latter makes assumptions about the chemical connectivity, the persistent homology approach is unbiased in this sense. Furthermore, while traditional ring analyses are generally meant for categorizing the number of atoms in the rings (a measure also directly obtained by persistent homology), the birth and death of loops conveniently provide quantitative information on both size and shape of the atomic rings.

Generally, the application of persistent homology to amorphous MRO structures is challenging, as the definition of characteristic regions (i.e., groups of loops) adopted in the original studies (*20*–*23*, *31*) is not unique; that is, they use an “optimal cycle” for the geometric characterizations. Here, we introduce a self-consistent and rigorous definition of characteristic regions by separating them according to the number of contained atoms (see below for definitions). This methodology offers an intuitive understanding of the underlying data (here, glass structure). Moreover, the interpretation of persistence diagrams has previously only been qualitative and relied on visual inspections, making comparisons among different structures highly complex. We propose to overcome these limitations by rigorously categorizing the persistence diagram features according to their structure, e.g., number of atoms and geometry, and by applying the accumulated persistence function (APF) (*26*)

$$\text{APF}(t)=\sum_{i:m_{i}\leq t}(d_{i}-b_{i})$$ (1)

where *b_i_* and *d_i_* are the birth and death times of point *i* in the persistence diagram, and *m_i_* = (*b_i_* + *d_i_*)/2 is the average length (i.e., “mean age”) of point *i*. With this terminology, a given mean age is equivalent for two points if the points are lying orthogonally to the same point on the diagonal of the persistence diagram. We may also define the “lifetime” of point *i* as *l_i_* = *d_i_* – *b_i_*, which measures how close neighboring atoms are in a loop and weighs it against the separation of the most distant atoms. That is, if local atoms are in proximity, then the loop will be born early and, similarly, if the most distant atoms are in proximity, then the loop will die early. This corresponds to a short lifetime. In other words, to obtain a point with long lifetime, nearby atoms should be close in space but well separated from the most distant atoms.

In detail, the APF is a cumulative sum of all points in the persistence diagram weighted by their lifetime. This means that the APF characterizes the persistence diagram completely and is especially well suited for data where “short-lived” loops or voids (as found in glasses) are real structural features and not noise. The function has previously been applied within the field of spatial statistics (*26*), but here, we apply it to enable direct quantitative comparison of multiple persistence diagrams in a single plot through the shape of the obtained curves. As it will be shown here, the approach is a simple, yet powerful, alternative to more computationally demanding methods for comparison of persistence diagrams, such as those based on machine learning. Furthermore, to deconvolute the contributions of structural MRO features to the FSDP, we introduce the *S*_PH_ function, which has previously been used on glassy SiO_2_ (*21*) and two amorphous ices (*23*)

$$S_{\text{PH},A_{k}}(Q)=\frac{1}{|A_{k}|}\sum_{(b│k,d_{i})\in A_{k}}\delta (Q-\frac{2\pi }{l(d_{i})})$$ (2)

where *Q* is the scattering vector, |*A_k_*| is the number of elements of a characteristic region (mathematically a subset) *A_k_*, and δ is the Dirac delta distribution. $l(d_{i})=2\sqrt{d_{i}+r_{O}^{2}}$ is the diameter of oxygen atoms (where $r_{O}=1.289\;\overset{{}^{\circ}}{A}$ is the radius of oxygen) at the time of death of loop *i*, which is the diameter at which the union of balls fills in the loop, largely corresponding to the “mean” diameter of loop *i*. This nonintuitive definition of the loop diameter originates from how increments of radius are defined (*27*). Last, we note that the function of Eq. 2 originates from the definition of a reciprocal space, where a repeating real-space distance, *l*, is equivalent to a magnitude of *Q* = 2π/*l* in reciprocal space (so-called *Q* space). By using the diameter of oxygen at the time of death as the real-space distance descriptor of the loop size, we simply sum up the reciprocal space contributions to *S*_PH_ of all loops using a summation sign and the Dirac delta distribution. We use the radius of oxygen, as oxygen has the largest radius of the three types of atoms, and because it is the most abundant element in both the glass compositions as well as in the identified loops.

In this work, we use classical molecular dynamics simulations to generate structures of *x*Na_2_O-(100 − *x*)SiO_2_ (0 ≤ *x* ≤ 40) glasses (*32*). By using the persistent homology-based methodology, we categorize MRO structures and their contributions to the FSDP of these sodium silicates, showing good agreement between the FSDP position computed directly from the RDF and that from persistent homology. We chose sodium silicates as a model system because of its well-studied structure (*12*, *13*, *33*) and because it is a simple representation of the structural features found in complex silicate glasses, including volumetric compaction and depolymerization of the silicate network upon modifier addition (*34*). In addition, sodium silicate glasses offer a rich system to investigate the relationship between network topology, persistence diagrams, and the FSDP.

## RESULTS

### Molecular dynamics simulations of glass structure

As shown in figs. S1 and S2, we generally find good agreement between the simulated and experimental neutron structure factors and *Q^n^* speciation (where *Q^n^* denotes SiO_4_ tetrahedra with *n* number of bridging oxygens) (*33*, *35*–*38*). We note that the minor differences between experiments and simulations do not affect the conclusions of this study. This is because we compare the structural information obtained from the simulated RDF with that from the persistent homology analysis; i.e., both are based on trajectory information from the same molecular dynamics simulations. Generally, the incorporation of Na_2_O into the silica network causes the fully connected structure to become depolymerized as nonbridging oxygens are formed and internal voids are filled by Na^+^. The latter is experimentally observed as an increase in the packing factor (*34*). This is also seen in the present simulations as presented in fig. S3 (*39*). Similarly, the ring size distribution of the structure is seen to notably broaden upon increasing Na_2_O content (fig. S4).

**Fig. 2:**
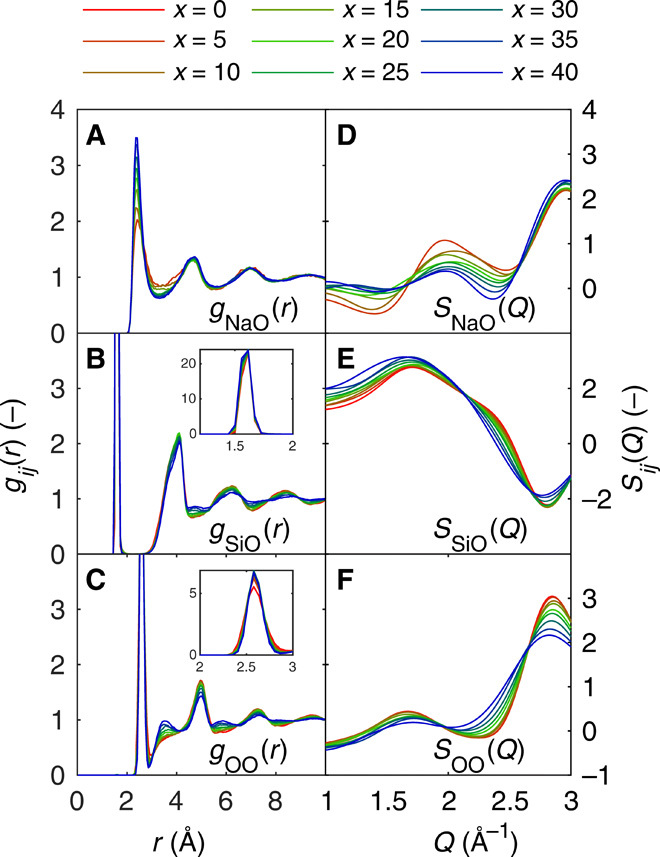
Glass structure analyses from molecular dynamics simulations. Partial RDFs [*g_ij_*(*r*), left column] as well as partial Faber-Ziman structure factors [*S_ij_*(*Q*), right column] for (**A** and **D**) NaO, (**B** and **E**) SiO, and (**C** and **F**) OO atomic pairs. Noticeable composition variation with the Na_2_O content is observed for all presented correlations. Insets in (B) and (C) show enlargements of the first peak of each *g_ij_*(*r*).

Figure 2 shows the partial RDFs [*g_ij_*(*r*), left column] and partial Faber-Ziman structure factors [*S_ij_*(*Q*), right column] for the (A and D) NaO, (B and E) SiO, and (C and F) OO atomic pairs. The remaining atomic pairs (NaNa, NaSi, and SiSi) are presented in fig. S5. For the Na RDFs, only minor changes are noted upon increasing sodium content, except for a slight decrease of position and intensity of the first peak in the NaNa RDF (fig. S5A) and an increase in the sharpness of the first peak (~2.3 Å) of the NaO RDF (Fig. 2A). For the NaSi (fig. S5B) and SiSi RDFs (fig. S5C), only small changes are found, while the SiO (Fig. 2B) and OO RDFs (Fig. 2C) show the largest variation with increasing sodium content. For SiO, this is noted as a slight decrease in intensity of the second peak (~4.0 Å) and an increase in intensity of a small peak at ~4.8 Å. Similarly, the OO RDF features increasing intensities for the peaks at 2.6, 3.6, and 5.9 Å, while decreasing intensities are noted for peaks at 5.0 and 7.3 Å. The remaining peaks at larger separation of the SiO and OO RDFs exhibit general broadening with increasing Na_2_O content. Overall, these data suggest that, primarily, the silicate backbone of the glass is affected by the addition of Na_2_O.

### Generation of persistence diagrams

Next, these simulated glass structures are subjected to the topological data analysis. In previous works on persistent homology and glasses (*21*, *23*), nonunique definitions of the characteristic regions with limited structural interpretations were provided. Here, we adopt rigid geometry-based definitions of the presented characteristic regions (*C*). We define *C*_L_ as long loops consisting of ≥5 atoms, which are found to be large structures of mainly oxygen (67 to 82%), with remaining atoms being both Si and Na. *C*_3_ and *C*_4_ are defined as subloops of *C*_L_, consisting of exactly three and four atoms, respectively. With these definitions, we are left with 1500 to 2500 unaccounted loops (∼20% of all loops), which we denote as the remaining three- and four-membered loops (*C*_R_). In addition, we define *C*_T_ as the square [−∞, 0.45] × [−∞, 0.45] in the persistence diagram primarily consisting of triangular O-Si-O loops, hence only indicating local structure within the SiO_4_ tetrahedron. We note that *C*_T_ holds loops from both *C*_3_, *C*_4_, and *C*_R_, yet we only use *C*_T_ to exclude short-range order points of the persistence diagram in the *S*_PH_ analysis. The procedure for obtaining a persistence diagram has been illustrated in Fig. 1, which is based on a molecular dynamics simulation of the 20Na_2_O-80SiO_2_ glass at 300 K. Sketches of the characteristic regions embedded in the structure and the corresponding persistence diagram of these loops are also shown in Fig. 1. The defined regions have different attributes owing to the different structural characteristics. *C*_L_ is where loops are born early and have a long lifetime (close to the vertical axis). These are chains, for which the connected atoms are in spatial proximity (e.g., covalent Si-O structures or space-filling Na atoms at higher Na_2_O contents). Furthermore, the large lifetime of *C*_L_ indicates a large separation for the most distant atoms of the loops, typically found on opposite side of the loops. *C*_4_ is where loops have varying birth and death times. These are primarily ascribed to packed structures of oxygen and sodium atoms. *C*_3_ and *C*_R_ have varying birth times and short lifetimes (i.e. they are positioned close the diagonal). These are typically similar in composition to *C*_4_ but often more separated in space as seen by the later birth time. Last, *C*_T_ has both low birth time and low death time (close to the origin), emerging from the intra-tetrahedral O-Si-O structure.

**Fig. 3:**
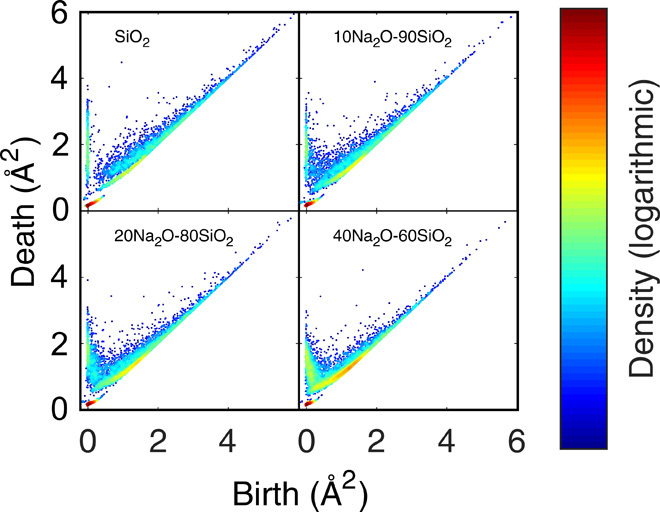
Persistence diagrams of loops of selected sodium silicate glasses. Persistence diagrams for structures at 300 K with *x* = {0,10,20,40} in the *x*Na_2_O-(100 − *x*)SiO_2_ system.

The topological implication of the sodium incorporation in the silica network is shown in Fig. 3, where the characteristic regions are observed to gradually change upon Na_2_O addition (the remaining persistence diagrams are shown in fig. S6). The number of loops in the low–birth time regions of *C*_L_, *C*_3_, and *C*_4_ increases as a result of Na_2_O addition (Fig. 3). We analyzed these loops and found them to be primarily oxygen (∼75% in average, decreasing with increasing sodium content) with smaller amounts of sodium and silicon. The decreasing birth time of these loops is due to the denser packing of oxygen atoms around sodium cations in the structure, providing shorter separation distances, in agreement with the partial RDFs (Fig. 2, A to C, and fig. S5, A to C). Such decrease of interatomic separation will decrease both birth and death times. The lifetime of *C*_L_ loops is also found to decrease with increasing Na_2_O content, as closed Si-O loops are expected to occur less frequently with increasing depolymerization of silicon-oxygen structures (fig. S2) and void filling by Na^+^ ions. Generally, the growth of the *C*_L_, *C*_3_, and *C*_4_ regions with increasing Na_2_O content is expected, given the increasing number of new structural features of the modified glasses, yet it demonstrates how persistent homology may be used to discover new MRO features in amorphous materials.

### Quantitative comparison of persistence diagrams

**Fig. 4:**
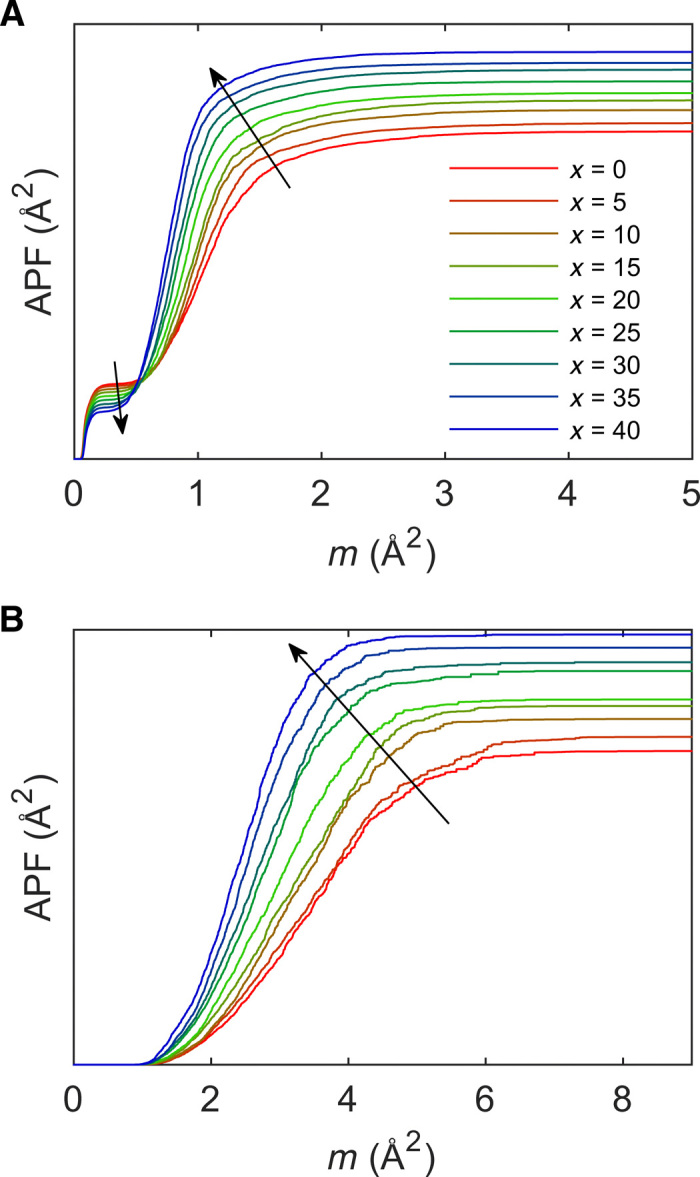
Quantitative analysis of persistence diagrams using the APF. The APF is presented for (**A**) loops and (**B**) voids as calculated from Eq. 1 for glasses *x* = {0,5,10,15,20,25,30,35,40} in the *x*Na_2_O-(100 − *x*)SiO_2_ system showing noticeable differences in MRO of the studied glasses. Arrows indicate tendency with increasing Na_2_O content.

While persistent homology is a powerful tool for studying amorphous structures, comparisons between persistence diagrams (as in Fig. 3) have previously been qualitative (*21*). To resolve this, we here compute the APF function (Eq. 1) for both loops (Fig. 4A) and voids (Fig. 4B) of the studied glasses. Figure 4A essentially compiles the information of Fig. 3 and fig. S6 into a single graph, by accumulating lifetimes as a function of the mean age, *m* = (*b* + *d*)/2, enabling quantitative comparisons. The shape of the APF curves is qualitatively similar for all the compositions, with an initial inflection point at *m* ∼0.25 Å^2^ and a following critical point where the APF intensity decreases with increasing Na_2_O content. As *m* increases further, the APF rises steeply to a maximum value, which is found at smaller *m* values for increasing Na_2_O content. We furthermore note that this maximum value increases with increasing Na_2_O content. The first inflection point at *m* ∼0.25 Å^2^ may be explained by differences in *C*_T_ related to the reduced number of Si atoms in the simulation, as this region primarily originates from the intra-tetrahedral three-atom O-Si-O structure (see Fig. 1 for a sketch of this structure). In contrast, we ascribe the second steep increase in the APF with *m* to the growth of *C*_L_, *C*_3_, and *C*_4_. These regions primarily consist of loops with all three atom types, yet with a majority of oxygen. Hence, the steep increase is due to an increased number of loops and not an increased lifetime of loops. Another important observation is the increase of the APF past the first inflection point, which occurs earlier with increasing Na_2_O content due to a decreased mean age of the loops. This corresponds to the data points (loops) moving closer to the origin of the plot in Fig. 3 with increasing Na_2_O content. These results for *C*_3_, *C*_4_, and *C*_R_ may be understood based on the effect of adding sodium, which fills structural voids of silicon and oxygen packing, hence creating new loops, consisting almost exclusively of O and Na. In turn, these are subloops of *C*_L_ but with shorter mean separation across the loop compared to pure O subloops and thus shorter death time and mean age. A similar decrease of mean age is also found for *C*_L_ as a result of the incorporation of sodium into the loops. This is in good agreement with the decreasing number of fully connected silicon tetrahedra (*Q*^4^ units, as seen in fig. S2) as well as with the changes of the partial RDFs of Fig. 2 (A to C) showing noticeable structural compaction.

Clear differences among the glasses are also noted in the APFs of the network voids (Fig. 4B). The corresponding persistence diagrams of voids, which have been used to construct Fig. 4B, are presented in fig. S7 and generally show slightly decreasing mean age and lifetime with increasing Na_2_O content. Structurally, this corresponds to smaller voids, in agreement with the increasing packing factor as both found experimentally (*34*) and in the present simulations (fig. S3).

While the ability of the APF to compare persistence diagrams is inherently useful, its additive nature also enables the contribution from each characteristic region to be deconvoluted. We provide an example of such analysis in fig. S8, finding *C*_L_ and *C*_4_ to only contribute to the second inflection of the APF, while *C*_3_ and *C*_R_ contribute to both inflections. Overall, we find that the combination of the persistence diagram and APF is a valuable tool for probing MRO and atomic packing in complex amorphous systems. That is, while visual, qualitative inspection of the persistence diagrams allows for some categorization of the different characteristic regions, the comparison of APFs offers a robust method for quantitatively analyzing differences in the contributions of the characteristic regions to the persistence diagram.

### Deconvoluting the FSDP by persistent homology

**Fig. 5:**
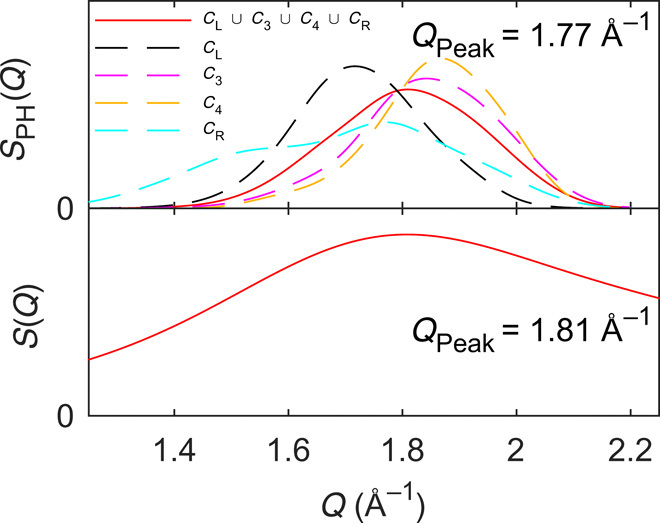
Deconvolution of the FSDP. Comparison between the *S*_PH_(*Q*) function calculated from Eq. 2 for all characteristic regions as well as their union and the structure factor [*S*(*Q*)], both shown for the 20Na_2_O-80SiO_2_ glass. Note that we exclude *C*_T_ from this analysis.

The obtained persistence diagrams also hold quantitative information about loop/ring sizes (as measured by the death time) that can be related with experimental observables, such as the FSDP. To do so, we here use Eq. 2 to calculate the *S*_PH_(*Q*) function of the characteristic regions of the persistence diagram for a 20Na_2_O-80SiO_2_ glass and compare the results with *S*(*Q*) (Fig. 5). The latter is computed by the Fourier transform of the RDF using a Lorch-type window function to reduce ripples at low *Q* values (*40*). Note that we exclude contributions of *C*_T_ (i.e., contributions of the intra-tetrahedral O-Si-O loops) from all *S*_PH_(*Q*) function analyses to restrict the analyses to MRO features and that we use equal scattering lengths for all atomic pairs when comparing *S*(*Q*) to *S*_PH_(*Q*). Overall, we observe substantial differences in peak position among the different characteristic regions (highest *Q* value for *C*_4_ and lowest for *C*_R_). A comparison of the *S*_PH_(*Q*) function of the union *A_k_* = *C*_L_∪*C*_3_∪*C*_4_∪*C*_R_ with the computed *S*(*Q*) (see Fig. 5) reveals excellent agreement of peak positions (1.77 and 1.81 Å^−1^, respectively), providing evidence for the applicability of this approach to understand the origin of the FSDP.

**Fig. 6:**
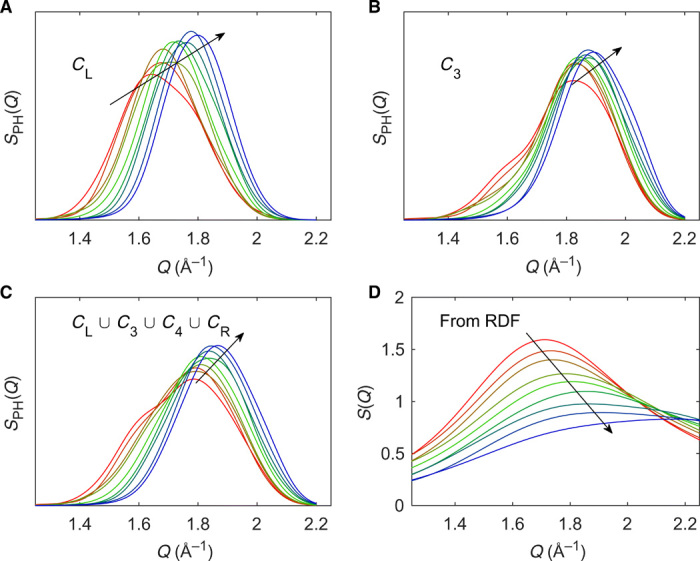
Composition-dependent changes in the contributions of various characteristic regions on the FSDP. A comparison between the *S*_PH_(*Q*) function as computed from different characteristic regions; (**A**) *C*_L_, (**B**) *C*_3_, (**C**) the union *C*_L_∪*C*_3_∪*C*_4_∪*C*_R_ of the persistence diagrams, and (**D**) the FSDP of the structure factor [*S*(*Q*)] obtained from the RDFs of structures from molecular dynamics simulations. The color coding is similar to Fig. 4, and arrows indicate tendency with increasing Na_2_O content.

To further understand the contributions to the FSDP, we calculate the *S*_PH_(*Q*) function for the characteristic regions *C*_L_, *C*_3_, *C*_4_, and *C*_R_ as well as the union of these for all the studied glasses in the *x*Na_2_O-(100 − *x*)SiO_2_ series. The *S*_PH_(*Q*) functions of *C*_L_, *C*_3_, and *C*_L_∪*C*_3_∪*C*_4_∪*C*_R_ are shown in Fig. 6 (A to C), while the remaining ones are shown in fig. S9. The peak positions of the *S*_PH_(*Q*) function for *C*_L_ (Fig. 6A), *C*_3_ (Fig. 6B), and *C*_L_∪*C*_3_∪*C*_4_∪*C*_R_ (Fig. 6C) are all found to increase with increasing Na_2_O content. Comparing these *S*_PH_(*Q*) functions with the computed *S*(*Q*) in Fig. 6D, we observe the same compositional dependence (increasing *Q* position with increasing sodium content), thus confirming the applicability of the proposed method to understand the FSDP. We directly compare the maximum position of *S*(*Q*) to the weighted average of the various *S*_PH_(*Q*) functions in fig. S10, finding the best agreement for *C*_4_ and the union *C*_L_∪*C*_3_∪*C*_4_∪*C*_R_ (fig. S10, C and E, respectively). This highlights the need for considering all characteristic regions in the description of the FSDP. We note some intensity differences between *S*_PH_(*Q*) functions and *S*(*Q*) and ascribe these to how *S*(*Q*), unlike *S*_PH_(*Q*), incorporates scattering interference contributions. This is supported by the work of Du and Corrales (*12*, *13*), which found the FSDP intensity of three alkali (Li, Na, and K) silicates to mainly vary because of the difference in their neutron scattering lengths and not because of differences in their MRO. That is, the FSDP intensity of lithium silicate (lithium has a negative neutron scattering length) varies inconsiderably with increasing Li_2_O content, while the FSDP intensities of sodium and potassium (both have positive neutron scattering lengths of similar magnitude) silicates show a substantial and similar decrease upon modifier addition. Similarly, the position of the FSDP has been found to only vary unnotably when changing the alkali ion (*13*), highlighting the pronounced effect of scattering interference in the obtained FSDP intensity from Fourier transform of the RDF.

On the basis of the above analysis, we conclude that the compositional dependence of the FSDP in sodium silicate glasses is related to all of the studied characteristic regions but with more pronounced contributions from large (>3 atoms) loops. Given the major abundance of oxygen in all these regions and the large radii of O and Na compared to Si, we ascribe the FSDP in the present case to originate from structural features of oxygen/sodium packing. This result is consistent with the OO partial structure factor (Fig. 2F), which is the only pair correlation to feature the *Q* correlation seen in Fig. 6D. The structural origin of these observations is likely ascribed to the packing of Na^+^ in the voids of the silicate backbone structure. Within the structure, the cationic sodium provides additional bonding between otherwise repulsive parts of the Si-O network. This reduces the distance between oxygen atoms due to their shared coordination to Na^+^ (see Fig. 1 for a schematic illustration of such packing). This effect becomes enhanced at higher sodium content. Similarly to the oxygen-oxygen correlation, the NaO partial structure factor (Fig. 2D) also shows a shift and a decrease in intensity with increasing Na_2_O content. Furthermore, these considerations are in good agreement with the partial OO RDF (Fig. 2C), showing a clear growth of the second peak at ~3.5 Å and a related decrease in intensity of the third peak at ~5 Å upon increasing Na_2_O content. Such compaction of the structure with increasing sodium content is also seen from the increase in intensity of the first peak in the NaO correlation (Fig. 2A) and the decrease in correlation length of the NaNa correlation (fig. S5A). We note that these results agree with the decrease in the mean age (i.e., atoms are less separated on average) of the second inflection point of the APF (Fig. 4A), the steeper slope during the APF increase (Fig. 4A), and the decreasing void size (Fig. 4B). Last, the reduced separation is in agreement with the increasing *Q* values of the FSDP in inverse space upon increasing Na_2_O content (Fig. 6D).

## DISCUSSION

We first compare the present persistent homology approach for analyzing the FSDP with other models. The model of Shi *et al*. (*3*) relates the silica ring size distributions from silicate crystals to the glassy FSDP. In comparison, the present approach has the advantage of not having any crystallography-based assumptions regarding the diameter of the rings. Similarly, the present approach allows for more direct structural deconvolution than the questionable prepeak in the concentration-concentration structure factor (*17*, *18*) considered in Elliot’s void model (*6*). Last, while the recent model of Shi and Tanaka (*4*) offers new insights into the contribution of the tetrahedral unit to the FSDP, the present approach is more widely applicable also to nontetrahedral structures. Several other approaches for explaining the FSDP exist, mainly based on describing the FSDP through the size and distribution of nanovoids in the studied materials (*2*, *41*). Such models generally rely on first defining the voids and then their size, typically requiring a number of assumptions on, e.g., chemical bonding. The persistent homology approach greatly reduces the number of such assumptions (and thus the potential bias) to the atomic radii and inherently includes all atom types in the computation, with a description of the loop/ring size through the death time. As such, the advantage of our developed persistent homology-based methodology lies in its applicability to any amorphous system, as it only requires atomic trajectory and radii information, without requirements of prior knowledge of chemical bonding.

In comparison to the previous work on persistent homology, this work considerably expands the number of amorphous structures studied by the *S*_PH_ function (from 3 to 12) (*21*, *23*) and provides the first rigorous test of the methodology for a systematic composition variation in a model glass series. As such, we have generalized the use of persistent homology to deconvolute the contribution of various MRO features to the FSDP in oxide glasses. By introducing self-consistent rigorous geometrical definitions of the structural units compared to previous works, we decode the persistence diagrams into structural features with simple geometrical origin. In addition, by implementing the so-called APF as a quantitative analysis tool for amorphous structures, we are able to decipher how the structural features identified by persistent homology evolve as a function of, e.g., time, stress, or composition. Consequently, we believe that this approach may lead to the settlement of the long-lasting debate regarding the origin of the FSDP in glassy systems. More generally, it can be applied to analyze and categorize molecular dynamics data and reveal hidden MRO in various amorphous materials.

## METHODS

Sodium silicate glass structures (3000 atoms) were simulated using the potential of Teter (*32*, *42*), following the quenching procedure in (*43*). Comparisons of experimental and simulated neutron *S*(*Q*) and *Q^n^* speciation are shown in figs. S1 and S2, respectively. We note a slight discrepancy in the FSDP between experiments and molecular dynamics. However, this has no influence on conclusions of this work, which only deals with comparisons of the FSDP from the RDF and persistent homology, both based on the same molecular dynamics simulations. Furthermore, the compositional dependence of density and packing factor, as well as the Si-O ring size distribution, is shown in figs. S3 and S4. We generally find good agreement between experiments and simulations. We computed the partial radial distributions and structure factors functions as averages over 100 ps of simulation time. This was done to obtain partial NaNa RDFs of meaningful quality for the structures of lowest sodium concentration. We found negligible differences in the partial structure factors and the total FSDP position when comparing static and time-averaged structure factors.

Persistent homology analyses were conducted using the libraries Diode (*44*) and Dionysus 2 (*45*), which incorporate computation based on periodic boundary conditions. We have computed the presented persistent homology functions using both energy-minimized (i.e., inherent configuration) and unminimized structures of all glass compositions, showing negligible differences. All presented data are thus for unminimized structures. In the treatment of the atomic radii (*r*), we follow the approach in (*21*) to obtain *r*_O_ = 1.289 Å, *r*_Na_ = 1.070 Å, and *r*_Si_ = 0.326 Å.

## SUPPLEMENTARY MATERIALS

Supplementary material for this article is available at http://advances.sciencemag.org/cgi/content/full/6/37/eabc2320/DC1
